# A Systematic Study on the Processing Strategy in Femtosecond Laser Scribing via a Two-Temperature Model

**DOI:** 10.3390/ma16216895

**Published:** 2023-10-27

**Authors:** Rujia Wang, Yufeng Wang, Yong Yang, Shuowen Zhang, Yunfeng Liu, Jianhua Yao, Wenwu Zhang

**Affiliations:** 1 Ningbo Institute of Materials Technology and Engineering, Chinese Academy of Sciences, Ningbo 315201, China; rujiawang@nimte.ac.cn (R.W.); wangyufeng@nimte.ac.cn (Y.W.); yangyong1994@nimte.ac.cn (Y.Y.); zhangsw_edu@163.com (S.Z.); 2 Zhejiang Key Laboratory of Aero Engine Extreme Manufacturing Technology, Ningbo 315201, China; 3 University of Chinese Academy of Sciences, Beijing 100049, China; 4 College of Mechanical Engineering, Zhejiang University of Technology, Hangzhou 310023, China; liuyf76@126.com (Y.L.); laser@zjut.edu.cn (J.Y.)

**Keywords:** laser beam machining, heat accumulation effect, ablation characteristics, process optimization, processing strategy

## Abstract

Balancing quality and productivity, especially deciding on the optimal matching strategy for multiple process parameters, is challenging in ultrashort laser processing. In this paper, an economical and new processing strategy was studied based on the laser scribing case. To reveal the temperature evolution under the combination of multiple process parameters in the laser scribing process, a two-temperature model involving a moving laser source was developed. The results indicated that the peak thermal equilibrium temperature between the electron and lattice increased with the increase in the laser fluence, and the temperature evolution at the initial position, influenced by subsequent pulses, was strongly associated with the overlap ratio. The thermal ablation effect was strongly enhanced with the increase in laser fluence. The groove morphology was controllable by selecting the overlap ratio at the same laser fluence. The removal volume per joule (i.e., energy utilization efficiency) and the removal volume per second (i.e., ablation efficiency) were introduced to analyze the ablation characteristics influenced by multiple process parameters. The law derived from statistical analysis is as follows; at the same laser fluence with the same overlap ratio, the energy utilization efficiency is insensitive to changes in the repetition rate, and the ablation efficiency increases as the repetition rate increases. As a result, a decision-making strategy for balancing quality and productivity was created.

## 1. Introduction

Ultrashort lasers gain advantages over long pulse lasers in high-precision machining, since the pulse duration is generally much shorter than the timescale for energy transfer to the lattice subsystem through electron–phonon coupling [1]. Currently, ultrashort laser processing has attracted much attention in fields such as biomedical [2], electronics manufacturing [3], miniaturization of photonic devices [4], laser-inscribed waveguides for realizing high-power ultrafast lasers with GHz repetition rates [5,6], aerospace manufacturing [7,8], etc.

For competitive industries, the demand for high processing throughput remains a key issue to be solved. Increasing the pulse energy or the number of pulses per unit time, i.e., the repetition rate, is a common solution to boost productivity. At the same time, the heat accumulation effect has gradually attracted attention [9]. For the fabrication of optical waveguides, the heat accumulation facilitated the formation of a symmetrical guiding cross-section [10]. Wang et al. reported a method for the rapid preparation of graphitized hierarchically porous carbon sheets based on the heat accumulation effect via a high-repetition picosecond laser [11]. When it comes to the processing of metals, however, the heat accumulation effect is regarded as detrimental because of severe melting behavior and significantly decreased accuracy. Nonetheless, as confirmed by Di Niso et al. [12], heat accumulation could considerably decrease the ablation threshold at a higher repetition rate for picosecond and femtosecond pulses, which is useful for enhancing the removal rate. Therefore, balancing quality and productivity in ultrashort laser processing, especially deciding on the optimal matching strategy for multiple process parameters, is quite challenging.

The literature has already reported several studies aiming to improve quality or productivity by optimizing the process parameters, in which a lot of research on the heat accumulation effect has been involved. Finger et al. [13] figured out that the presence of the heat accumulation effect stemmed from three aspects, i.e., subsequent pulses, subsequent scanning passes, and multiple spots during the machining process. The cause of the heat accumulation lies in the inappropriate time and space separation. Weber et al. studied the heat accumulation effect via an analytical solution, and the recommended laser power at a given repetition rate could be deduced [14]. Bauer et al. studied the heat accumulation effect in ultrashort laser ablation of metals and stated that there is a critical scanning speed that enables the transition of the ablation mechanism [15]. Zhang et al. analyzed the heat accumulation effect based on a three-dimensional heat conduction model and revealed that a high-speed scanning strategy contributed to a higher ablation efficiency [16].

In addition to the heat accumulation effect, improving the productivity of ultrashort laser processing is also an object of great interest. As discussed in the literature [17,18,19], the strategies of selecting an optimal beam spot, repetition rate, or laser fluence can be adopted to achieve the most efficient material removal based on the ablation model for short and ultrashort pulses. Moreover, other methods, including deep neural networks or machine learning [20,21,22], were adopted not only to establish the predictive model but also to reduce the time to optimize the process parameters. There seems to be a trade-off between ablation quality and productivity, which is a contradictory topic. For the time being, a better understanding of the underlying physical pictures and mechanisms is fundamental and necessary to solve such a problem in ultrashort pulse laser ablation. The two-temperature model (TTM) [23] has a great advantage in describing the non-equilibrium heat transfer between electron and lattice. The TTM coupled with molecular dynamics [24] and hydrodynamics [25] has become an important means to reveal the thermophysical pictures in the lattice subsystems.

In this paper, a decision-making mechanism for ultrashort laser processing, especially in the context of multiple process parameters, including pulse energy, repetition rate, scanning speed, and overlap ratio, was discussed. In Section 2, the materials and methods used in the investigation were introduced. The experiment of laser scribing on the nickel-based alloy was conducted to investigate the effects of the above process parameters on ablation quality and productivity. In Section 3, a two-temperature model involving a moving laser source was developed to study the temperature evolution under the action of multiple pulses. The morphological features at different laser fluences were compared, and the quantitative statistics of the ablation characteristics influenced by multiple process parameters were carried out. Finally, a processing strategy for balancing quality and productivity was developed. The experimental results and the simulation analysis provided a theoretical basis and technical support for the production scenarios, such as laser trepan drilling of cooling holes, laser cutting, laser engraving, laser polishing, etc.

## 2. Materials and Methods

### 2.1. Materials

The sample was a DZ411 nickel-based alloy block with a size of 10 mm × 20 mm × 2 mm. The alloy is a directionally solidified nickel-based casting superalloy with excellent hot corrosion resistance properties, which is widely used in the aviation field. The chemical composition of the DZ411 nickel-based alloy (in wt.%) is shown in Table 1.

### 2.2. Laser Machining System

**Figure 1:**
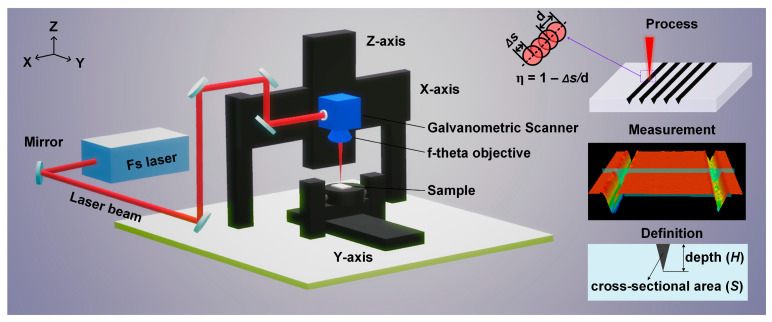
Schematic diagram of the laser machining system and morphological features for the scribed groove.

Figure 1 shows the schematic diagram of the laser machining system. A stable and reliable femtosecond pulsed fiber laser (HR-Femto-IR-200-35, Wuhan Huaray Precision Laser Co., Ltd., Wuhan, China) was used for the laser scribing. The laser system emitted 317 fs pulses with a maximum pulse energy of 200 μJ at 1030 nm. The maximum pulse repetition rate was 175 kHz. The beam quality factor M^2^ was approximately 1.3. The beam trajectory was controlled by a two-axis galvanometric scanner (SUPERSCAN IIE, RAYLASE, Wessling, Germany). The laser beam was focused on the surface of the samples by an f-theta objective (LINOS F-Theta-Ronar, Excelitas Technologies Corp., Waltham, MA, USA) of 167 mm focal length into a spot diameter of 70 μm with Gaussian energy distribution. The motion platform featured a resolution of 5 μm and a total travel distance (X/Y/Z) of 500 mm × 500 mm × 300 mm.

### 2.3. Experimental Arrangement

Before the experiments, the samples were immersed in a pure alcohol solution and cleaned by an ultrasonic cleaning machine (KQ2200DE, Kunshan Ultrasonic Instrument Co., Ltd., Kunshan, China) for 10 min. The arrangement of the laser scribing experiments is shown in Table 2. The overlap ratio can be calculated as

(1) $$\eta =1-\frac{∆s}{d},$$

and

(2) ∆s=v/f,

where ∆s is the distance between two adjacent pulses along the scan direction, d is the focus diameter, *v* is the scanning speed, and *f* is the repetition rate. The scan track is a straight line 1 mm in length, with 50 passes for each set of experimental parameters.

### 2.4. Characterization Analysis

The scribed samples were cleaned by the ultrasonic cleaning machine and dried with blowing air. The surface morphology of the scribed samples was observed by a scanning electron microscope (Sirion 200, FEI, Hillsboro, OR, USA). The depth and cross-sectional area of the scribed groove, as shown in Figure 1, were measured by a laser scanning confocal microscope (VK-X200K, KEYENCE, Osaka, Japan).

The other two parameters, including removal volume per joule ($V_{e}$) and removal volume per second ($V_{t}$), were defined as [16]

(3) $$V_{e}=\frac{Sv}{{NE}_{p}f},$$

and

(4) $$V_{t}=\frac{Sv}{N},$$

respectively. In the above equations, S is the cross-sectional area of the scribed groove, v is the scanning speed, N is the number of scans, $E_{p}$ is the pulse energy, and f is the repetition rate.

## 3. Results and Discussion

The effects of multiple process parameters, i.e., pulse energy, repetition rate, scanning speed, and overlap ratio, on the ablation quality and productivity, were investigated. Different laser fluences, ranging from 0.416 J/cm^2^ to 8.32 J/cm^2^, were applied on the sample surface by varying the pulse energy. The ablation threshold ($\phi_{th}$) of the DZ411 nickel-based alloy was approximately 0.252 J/cm^2^, as measured by the ablation threshold method [26]. Various scribed groove structures were inspected. The temperature evolution in the scribing process and ablation characteristics (depth, cross-sectional area, removal volume per joule, and removal volume per second) influenced by multiple process parameters were analyzed.

### 3.1. Morphology of Grooves Scribed by the Femtosecond Laser

**Figure 2:**
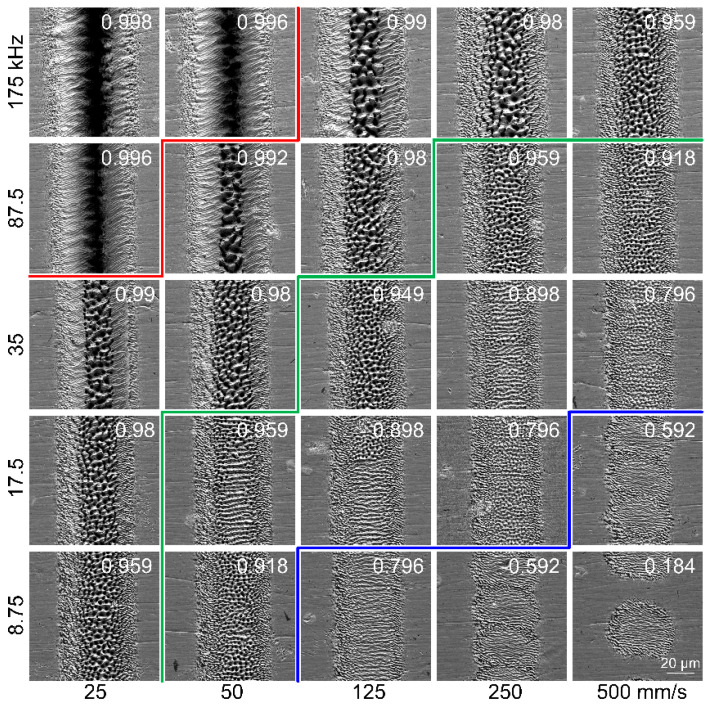
SEM images of the grooves at various combinations of scanning speed and repetition rate. The pulse energy is 8 μJ, and the peak laser fluence is 0.416 J/cm^2^. The number of scans is 50. The number in the upper right corner of each subgraph represents the overlap ratio. The red, green and blue lines in the figure are only used to distinguish transitions in the morphology of microgrooves.

Figure 2 shows the SEM images of the grooves at various combinations of scanning speed and repetition rate with a pulse energy of 8 μJ. The corresponding peak laser fluence is 0.416 J/cm^2^, which is approximately 1.65 times the ablation threshold of the sample. At the rim of the grooves, mild ablation occurred due to the lower laser fluence at the edge of the Gaussian beam, and the corresponding morphology was characterized by the presence of laser-induced periodic surface structures (LIPSS). In the view of electromagnetic energy deposition theory, the formation mechanism of LIPSS under ultrashort laser pulses is as follows; the lattice temperature distribution of the material surface resembled the electric field intensity distribution, which was periodically distributed on the material surface [27]. Material ablation occurred where the lattice temperature exceeded the evaporation temperature. As a result, LIPSS microstructures were formed on the material surface.

**Figure 3:**
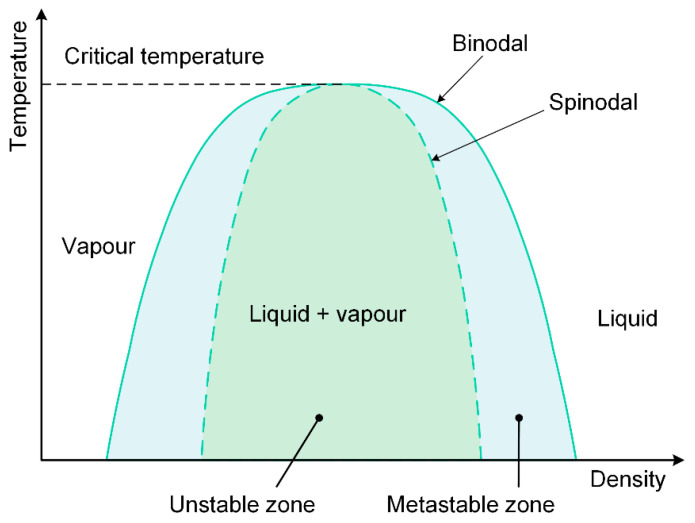
Density–temperature phase diagram of a liquid–vapor mixture (adapted from [33]).

The morphology at the central region of the groove, however, is quite different from that at the rim of the grooves, which is strongly correlated with the overlap ratio. When the overlap ratio equaled or exceeded 0.996, a strong thermal ablation occurred, and a groove with greater depth was obtained. When the overlap ratio fell between 0.959 and 0.996, peak-and-valley structures appeared at the central region of the groove. There is a lot of research on the formation mechanism of the observed structures [28,29,30]. Certain terminology was used in the following description for convenience, as shown in Figure 3, where the binodal line represents the one-phase fluid becoming metastable and the spinodal line is the metastability limit. At a higher overlap ratio, the surface material can be rapidly heated due to the prominent heat accumulation, although the laser fluence provided is close to the ablation threshold. This sufficiently led to the material entering deep into the metastable zone close to the spinodal line; homogeneous bubble nucleation might occur, and as a result, the irradiated material was discretized into a mixture of gas and liquid [31]. The gas bubbles quickly broke away from the surface of the superheating material, and as the liquid material cooled and solidified rapidly, the peak-and-valley structures were formed. It is indicated that phase explosion [32] may be responsible for the appearance of these peak-and-valley structures.

When the overlap ratio fell between 0.796 and 0.959, the morphology at the central region of the groove manifested itself as the coexistence of two kinds of microstructures, namely the striped structures and the peak-and-valley structures. When the overlap ratio fell below 0.796, the morphology at the central region of the groove was completely striped structures. With a further decrease in the overlap ratio, e.g., 0.592, the groove morphology became discontinuous.

It should be noted that the striped structures, featuring a larger spacing, are quite different from the LIPSS structures at the rim of the grooves. With the decrease in the overlap ratio, the eruption of boiling liquid material was suppressed due to the weakened heat accumulation effect, and a relatively gentle hydrodynamic process occurred, e.g., wavy phenomena such as capillarity waves [34], which may be responsible for the formation of the striped structures.

**Figure 4:**
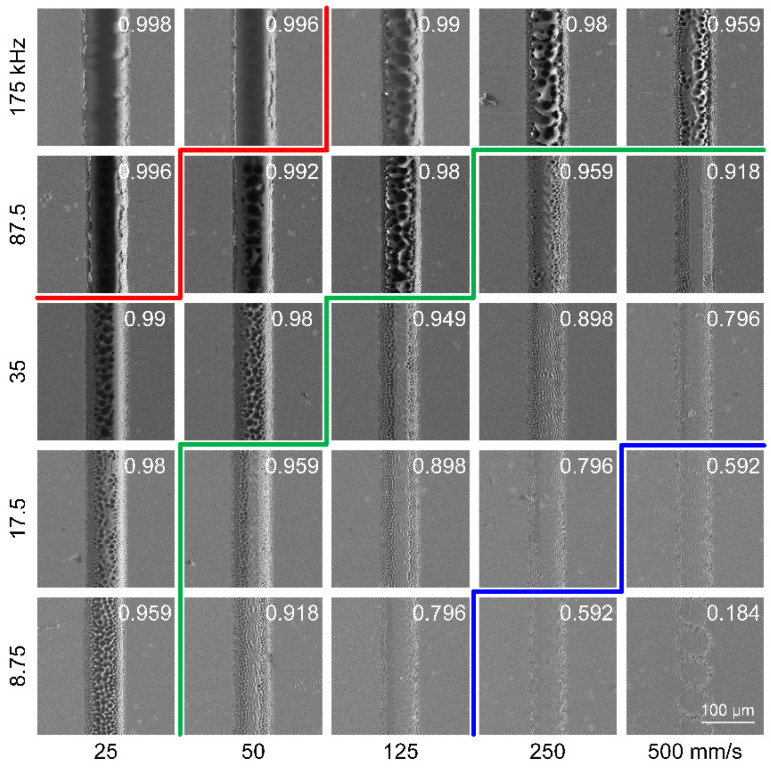
SEM images of the grooves at various combinations of scanning speed and repetition rate. The pulse energy is 40 μJ, and the peak laser fluence is 2.08 J/cm^2^. The number of scans is 50. The number in the upper right corner of each subgraph represents the overlap ratio. The red, green and blue lines in the figure are only used to distinguish transitions in the morphology of microgrooves.

**Figure 5:**
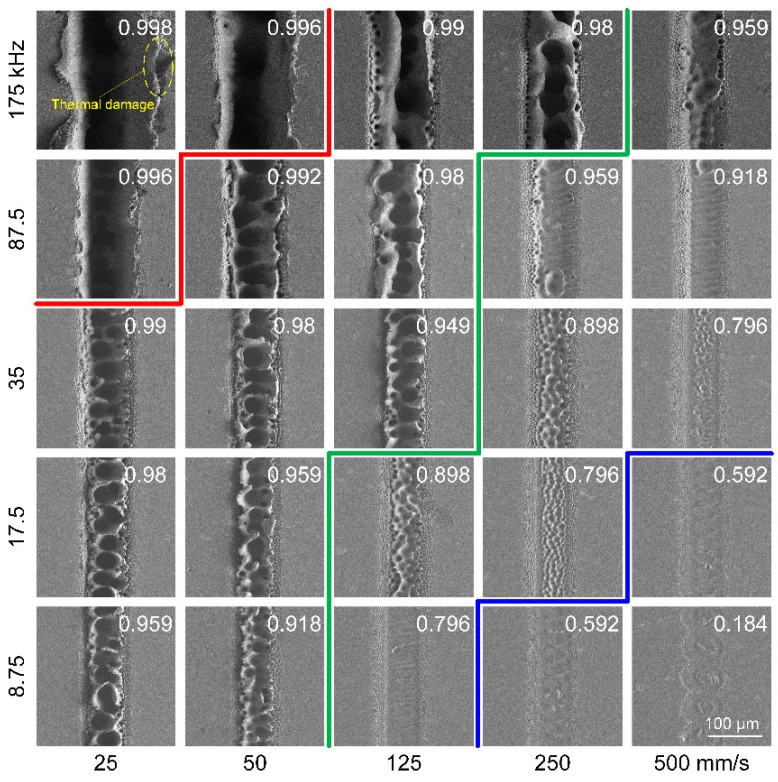
SEM images of the grooves at various combinations of scanning speed and repetition rate. The pulse energy is 160 μJ, and the peak laser fluence is 8.32 J/cm^2^. The number of scans is 50. The number in the upper right corner of each subgraph represents the overlap ratio. The red, green and blue lines in the figure are only used to distinguish transitions in the morphology of microgrooves.

Figure 4 and Figure 5 show the SEM images of the grooves at various combinations of scanning speed and repetition rate with a pulse energy of 40 μJ (2.08 J/cm^2^) and 160 μJ (8.32 J/cm^2^), respectively. The LIPSS structures at the rim of the grooves ceased to exist. Violent ablation occurred at such laser fluences, especially when a combination of a high repetition rate and a low scanning speed was used, and severe thermal damage appeared at the rim of the scribed groove. One could still observe a strong correlation between the morphological feature and the overlap ratio. A deep ablation depth was obtained when the overlap ratio equaled or exceeded 0.996. However, as the overlap ratio decreased, the morphology of the groove was significantly different.

For the case of 40 μJ (2.08 J/cm^2^), the sputtering pits with inhomogeneous voids were distributed in a disorderly manner at the central region of the groove when the overlap ratio fell between 0.959 and 0.996. It can be assumed that a large number of bubbles were initially formed under the irradiation of high laser fluences, and after further coalescence and coarsening, large bubbles increased and small bubbles decreased. As these bubbles escaped and the molten layer resolidified, the morphology was presented as sputtering pits. When the overlap ratio fell between 0.796 and 0.959, the morphology at the central region of the groove entered a transitional stage. When the overlap ratio was close to 0.959, smaller voids were distributed in the scribed grooves. The thermal effect was suppressed due to a further decrease in the overlap ratio, i.e., approaching 0.796, and the flow of the material in the liquid phase occurred and contributed to a flat and smooth surface at the central region of the grooves. When the overlap ratio was further reduced to 0.592, the grooves became discontinuous, with wavy edges at the rim of the grooves.

For the case of 160 μJ (8.32 J/cm^2^), one could observe a pronounced melting phenomenon, and crater-like structures were formed at the central region of the grooves when the overlap ratio fell between 0.959 and 0.996. At such a high laser fluence, a higher heating rate could be obtained, and superheating of the target material occurred. It seems that the formation of crater-like structures is related to the expansion and separation of the gas phase, which will be further discussed in the next section. When the overlap ratio fell between 0.796 and 0.959, the morphology at the central region of the grooves entered a transitional stage, where crater-like structures with smaller voids or a relatively flat surface were observed. When the overlap ratio was further reduced to 0.592, a pleated structure appeared at the central region of the grooves, which may be related to the hydrodynamic process during the laser scribing.

During the scanning of the laser, the accumulated laser fluence $H_{acc}$ can be defined as [35]

(5) $$H_{acc}=n_{s}\frac{2E_{P}}{\pi {w_{0}}^{2}}\underset{n=0}{\overset{n_{P}}{\sum }}\underset{m=0}{\overset{n_{H}}{\sum }}e^{-\frac{2[{\left(x-nd_{P}\right)}^{2}+{\left(y-md_{H}\right)}^{2}]}{{w_{0}}^{2}}},$$

(6) $$\eta_{x}=1-\frac{d_{P}}{2w_{0}},$$

and

(7) $$\eta_{y}=1-\frac{d_{H}}{2w_{0}},$$

where $n_{s}$ is the scan number, and $\eta_{x}$ and $\eta_{y}$ are the overlap ratio along the *x*-axis and *y*-axis directions, respectively. $w_{0}$ represents the focus radius, $d_{P}$ refers to the distance between two adjacent pulses along the *x*-axis, and $d_{H}$ refers to the distance between two adjacent scan paths along the *y*-axis. Since there is no overlap of the two scan paths involved in this paper, only the overlap ratio along the *x*-axis direction is considered.

**Figure 6:**
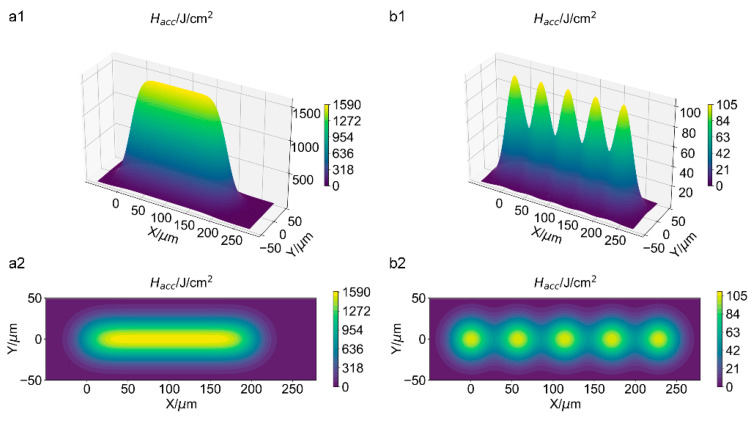
Analysis of the accumulated laser fluence H_acc_ at an overlap ratio of 0.959 (**a1**) and 0.184 (**b1**). The corresponding contour map for the accumulated laser fluence is (**a2**) and (**b2**), respectively. The pulse energy $E_{P}$ is 40 μJ, and the peak laser fluence is 2.08 J/cm^2^. The scan number $n_{s}$ is 50.

Figure 6 shows the analysis results of the accumulated laser fluence *H_acc_* at different overlap ratios. It indicates that the accumulated laser fluence *H_acc_* at a higher overlap ratio (η = 0.959) is evenly distributed along the *x*-axis, while *H_acc_* at a lower overlap ratio (*η* = 0.184) presents a periodic distribution with pronounced laser fluence maximums. The maximum *H_acc_* at a higher overlap ratio (*η* = 0.959) is about 15 times the value at a lower overlap ratio (*η* = 0.184). Experimentally, on the sample surface, the groove with a deep ablation depth and a smooth edge was obtained at a higher overlap ratio (*η* = 0.959), while the groove with a shallow depth and a wavy edge was obtained at a lower overlap ratio (*η* = 0.184). Therefore, it is shown that the groove morphology has a strong correspondence with the overlap ratio at the same laser fluence. To get more detailed effects of multiple process parameters on the groove morphology, the temperature evolution based on a theoretical analysis was investigated.

### 3.2. Temperature Evolution Revealed by the Theoretical Analysis

To further investigate the temperature evolution in the femtosecond laser scribing process at various combinations of repetition rate and scanning speed, the two-temperature model involving a moving laser source was developed. The heat transfers among photons, electrons, and lattices are governed by the following equations [36]:

(8) $$C_{e}(T_{e})\frac{\partial T_{e}}{\partial t}=\nabla \left(k_{e}(T_{e},T_{l})\nabla T_{e}\right)-g\left(T_{e}-T_{l}\right)+S(x,z,t)$$

and

(9) $$C_{l}\frac{\partial T_{l}}{\partial t}=\nabla \left(k_{l}\nabla T_{l}\right)+g\left(T_{e}-T_{l}\right),\;$$

where *C* is the heat capacity, *T* is the temperature, *k* is the thermal conductivity, and *g* is the electron–phonon coupling strength. The subscripts *e* and *l* represent the electron and lattice, respectively. $C_{e}$ and $k_{e}$ vary with the temperature, which reads as [37,38]

(10) $$C_{e}=\gamma T_{e}$$

and

(11) $$k_{e}=k_{e,0}\frac{T_{e}}{T_{l}},$$

respectively. γ represents the electronic heat capacity parameter, and $k_{e,0}$ represents the thermal conductivity of electrons at *T* = 273 K. To reduce the consumption of computing resources, the model was simplified to a two-dimensional model and was modeled in the *x*–*z* plane.

The laser heating source term, *S* (*x*, *z*, *t*), is expressed as [39]

(12) $$S\left(x,z,t\right)=\frac{\left(1-R\right)}{L_{p}}\frac{F}{\tau_{p}}\sqrt{\frac{4ln2}{\pi }}\frac{{w_{0}}^{2}}{w^{2}\left(z\right)}\exp\left[-\frac{z}{L_{p}}-4ln2{\left(\frac{t-2\tau_{p}}{\tau_{p}}\right)}^{2}\right]exp(\frac{-2{x_{s}}^{2}}{w^{2}\left(z\right)}).$$

(13) $$F=\frac{2E_{p}}{\pi {w_{0}}^{2}},$$

(14) $$w\left(z\right)=w_{0}\sqrt{1+\frac{z^{2}}{{z_{R}}^{2}}},$$

(15) $$z_{R}=\frac{\pi {w_{0}}^{2}}{\lambda },$$

and

(16) $$x_{s}=x-x_{0}-vt,$$

where *R* is the reflectance of the target material, $L_{p}$ is the optical absorption depth, F is the laser fluence, $\tau_{p}$ is the full width at half-maximum (FWHM) of the pulse duration with a value of 317 fs, and z represents the *z*-axis. The laser beam radius $w\left(z\right)$ varies along the *z*-axis, $z_{R}$ is the Rayleigh range, and the laser wavelength λ is 1030 nm. $x_{s}$ describes a moving laser source, $x_{0}$ is the initial position, v is the scanning speed, and x represents the *x*-axis. The physical parameters of nickel were used for the TTM simulation, as listed in Table 3. For the boundary conditions in this model, the thermally insulating boundary conditions on the free surface were used, and mass removal was not considered.

**Figure 7:**
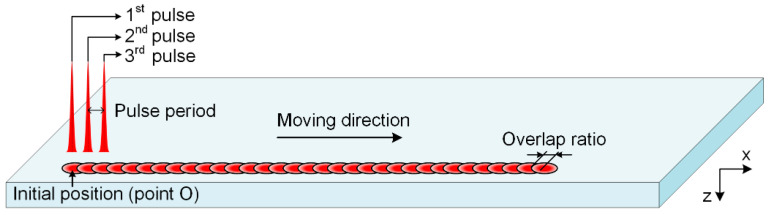
The schematic diagram of the simulation model.

Figure 7 shows the schematic diagram of the simulation model. To investigate the temperature evolution during the laser scribing process, three pulses were applied sequentially following various combinations of the repetition rate and scanning speed. The evolution of electron and lattice temperature under the action of different pulses at the initial position (i.e., point O) was investigated.

**Figure 8:**
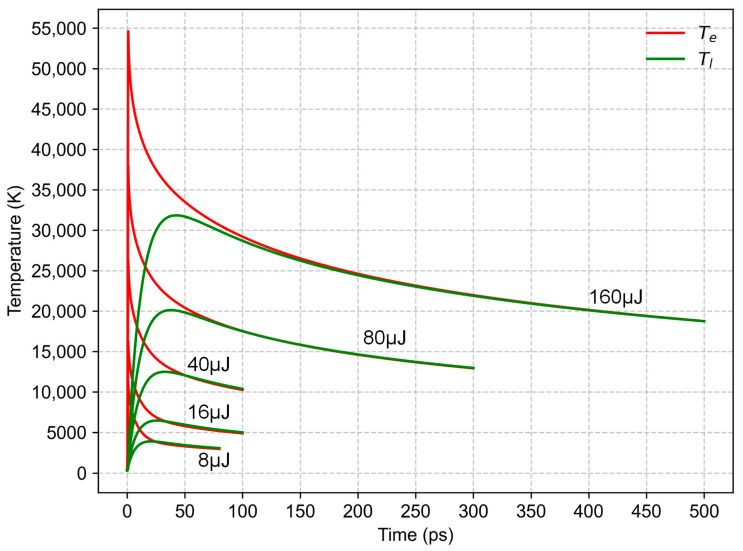
The electron–phonon relaxation process under the action of a single pulse at various pulse energies.

The electron–phonon relaxation process under the action of a single pulse at various pulse energies is shown in Figure 8. One can observe a rapid rise in the electron temperature, followed by a rise in the lattice temperature as electron energy is transferred to the lattice subsystem through electron–phonon coupling. It is shown that the peak thermal equilibrium temperature between the electron and lattice increases with the increase in the pulse energy. A higher heating rate can be obtained at high pulse energies (high laser fluences), leading to a rapid rise in the temperature of the electron and the lattice subsystem. The critical temperature of nickel is determined to be about 9470 K [42]. The peak thermal equilibrium temperature far exceeds the critical temperature of the target material when pulse energies of 40 μJ, 80 μJ, and 160 μJ are used, respectively. As the pulse energy increases, the time required to reach thermal equilibrium increases accordingly.

At low laser fluences, the corresponding pulse energy is 8 μJ or 16 μJ in this work, and the peak thermal equilibrium temperature is below the critical temperature of the target material (Figure 8). The thermodynamic pathway may follow the rules below: the material is heated to a hot liquid state, and as the material expands, the temperature of the material decreases. When the material system enters the metastable zone between the bimodal line and spinodal line, homogeneous bubble nucleation occurs, causing the heated material to be transformed into a mixture of gas and liquid. After the escape of gas bubbles and the subsequent solidification of the liquid layer, the peak-and-valley structures in Figure 2 were presented.

At high laser fluences, the corresponding pulse energy equals or exceeds 40 μJ in this work, and the peak thermal equilibrium temperature far exceeds the critical temperature of the target material (Figure 8). It is indicated that the material is rapidly heated to a supercritical state, and what happens next is of great interest. Two possible thermodynamic pathways, including fragmentation [43] and critical point phase separation [42], are invoked here. The specific mechanism strongly depends on the expansion rate of the materials. For the former, under fs irradiation, the buildup of a strong pressure within the material leads to a rapid thermal expansion and resultant breakage of the supercritical fluid. It corresponds to the fact that the material has already decomposed before entering the metastable zone. For the latter, with the expansion of the material, the temperature drops below the critical temperature, and after the material crosses the spinodal line and enters the unstable zone, the gas and the liquid phase separation occurs. From the two scenarios discussed above, it is reasonable to infer that there is a strong pressure release during laser scribing with high laser fluences. As the gas expands and the pressure is released, sputtering pit-like structures or crater-like structures are formed after the molten layer resolidifies.

**Figure 9:**
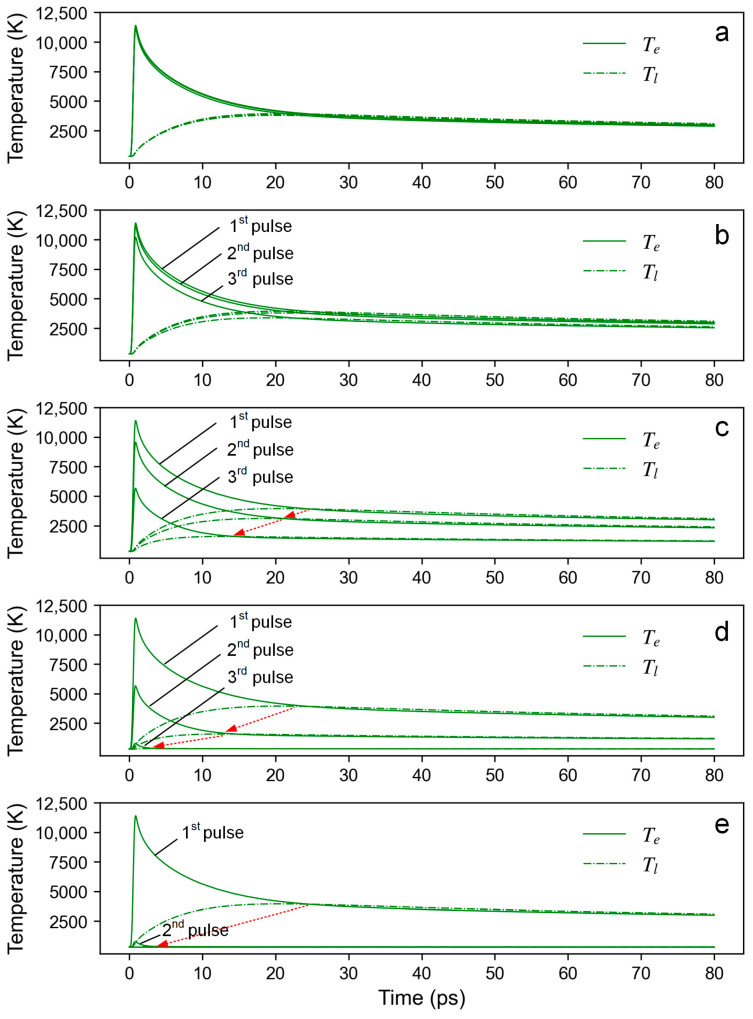
The electron–phonon relaxation process for the initial position (point O) under the action of three pulses at different scanning speeds: (**a**) 25 mm/s,η=0.959; (**b**) 50 mm/s, η=0.918; (**c**) 125 mm/s, η=0.796; (**d**) 250 mm/s, η=0.592; (**e**) 500 mm/s, η=0.184. The pulse energy is 8 μJ, and the peak laser fluence is 0.416 J/cm^2^. The repetition rate is 8.75 kHz. The red arrow represents a downward trend in thermal equilibrium temperature.

**Figure 10:**
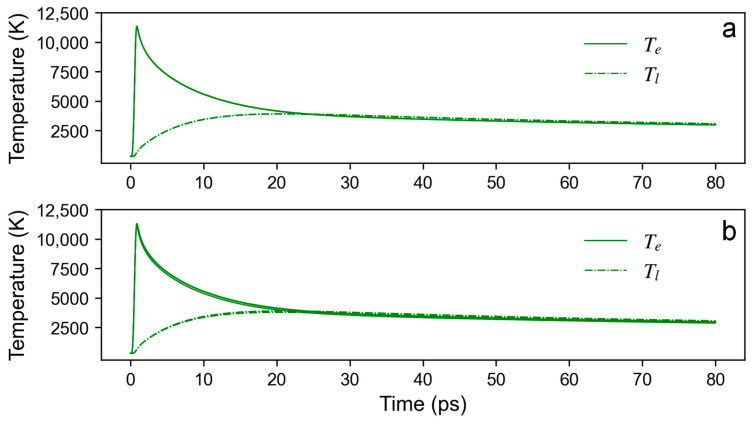
The electron–phonon relaxation process for the initial position (point O) under the action of three pulses at different scanning speeds: (**a**) 25 mm/s and (**b**) 500 mm/s. The pulse energy is 8 μJ, and the peak laser fluence is 0.416 J/cm^2^. The repetition rate is 175 kHz.

It should be noted that the temperature evolution is not only related to the pulse energy (laser fluence) but also to the overlap ratio. The pulse energy (laser fluence) determines the peak thermal equilibrium temperature that can be achieved, while the overlap ratio affects the strength of the heat accumulation effect. Figure 9 shows the electron–phonon relaxation process for the initial position under the action of three pulses at different scanning speeds, which reflects the thermal effect of the subsequent pulses on the initial position. The pulse energy is 8 μJ, and the peak laser fluence is 0.416 J/cm^2^. The repetition rate is 8.75 kHz. The overlap ratio drops from 0.959 at 25 mm/s to 0.184 at 500 mm/s. As shown in the simulation results, one can observe that the thermal effect of subsequent pulses on the temperature evolution at the initial position is significantly weakened when the overlap ratio falls to 0.796. Especially when the overlap ratio is reduced to 0.184, the thermal effect of subsequent pulses can be negligible. When the repetition rate is 175 kHz, the overlap ratio drops from 0.998 at 25 mm/s to 0.959 at 500 mm/s. It can be seen that the thermal effect of subsequent pulses on the temperature remains prominent even when a scanning speed of 500 mm/s is used, as is shown in Figure 10.

**Figure 11:**
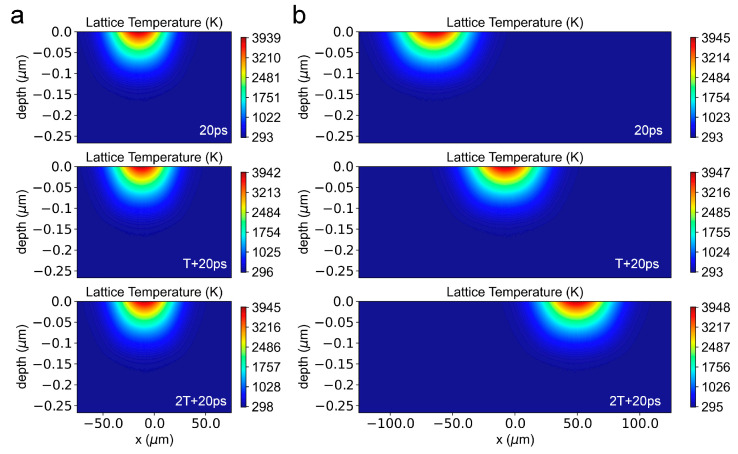
The lattice temperature evolution at scanning speeds of (**a**) 25 mm/s (η=0.959) and (**b**) 500 mm/s (η=0.184). The pulse energy is 8 μJ, and the peak laser fluence is 0.416 J/cm^2^. The repetition rate is 8.75 kHz.

Figure 11 illustrates the thermal effect induced by the overlap ratio. At a high overlap ratio (η=0.959), the laser source will stay near the initial position (x = −15 μm) for a long time, and as a result, a significant thermal effect can be reasonably inferred. Meanwhile, at a low overlap ratio (η=0.184), the thermal effect of subsequent pulses on the temperature evolution at the initial position (*x* = −65 μm) will greatly be suppressed due to the rapid departure of the laser source.

### 3.3. Ablation Characteristics Influenced by Multiple Process Parameters

**Figure 12:**
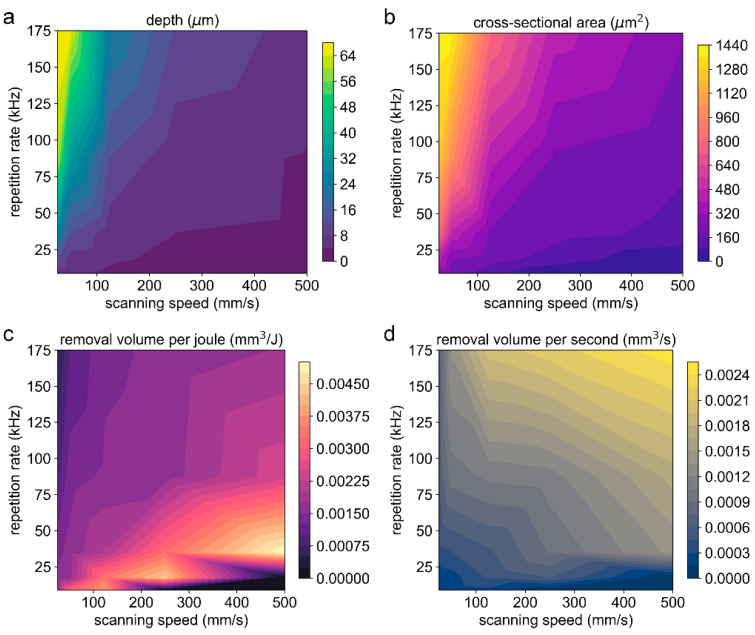
The contour map for the groove depth (**a**), cross-sectional area (**b**), removal volume per joule (**c**), and removal volume per second (**d**) at various combinations of the scanning speed and repetition rate. The pulse energy is 8 μJ, and the peak laser fluence is 0.416 J/cm^2^. The number of scans is 50.

In this section, the effects of multiple parameters on the ablation characteristics are discussed experimentally. Figure 12a–d shows the contour map for the groove depth, cross-sectional area, removal volume per joule (*V_e_*), and removal volume per second (*V_t_*), respectively, at various combinations of the scanning speed and repetition rate with a pulse energy of 8 μJ. It is shown that the maximum depth and the maximum cross-sectional area were obtained simultaneously at a combination of a high repetition rate and a low scanning speed. On the contrary, the minimum ones were obtained at the combination of a low repetition rate and a high scanning speed. However, the two parameters, including depth and cross-sectional area, cannot provide a more intuitive reference for the processing decision. For this reason, the removal volume per joule (*V_e_*) and the removal volume per second (*V_t_*) were introduced, where *V_e_* represents the energy utilization efficiency and *V_t_* the ablation efficiency.

**Figure 13:**
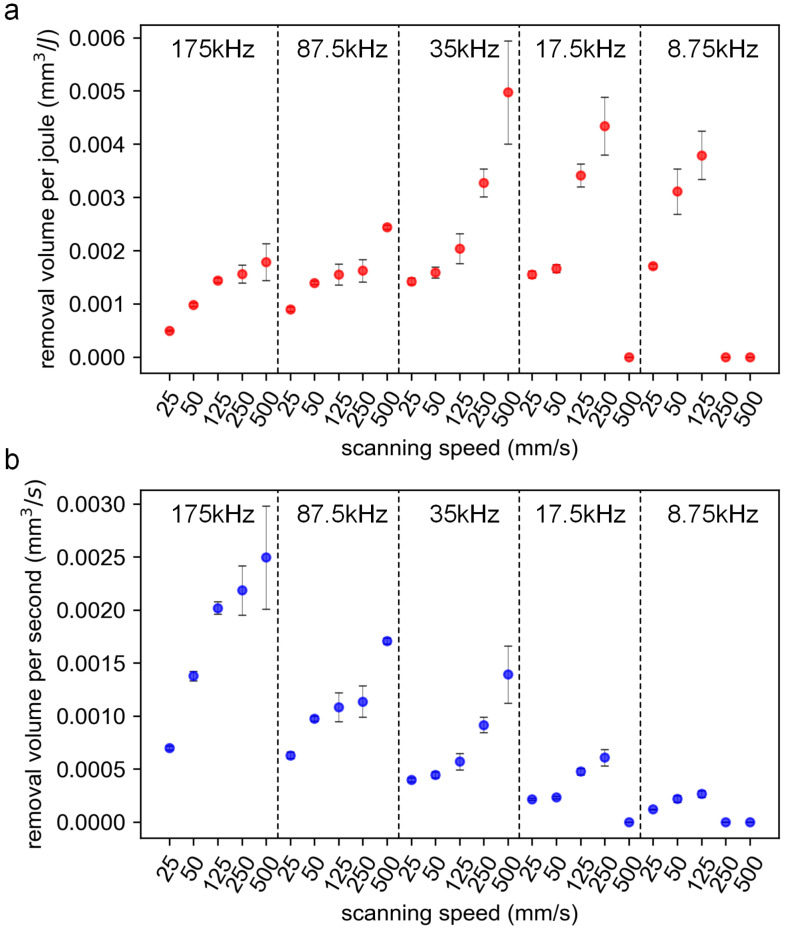
The removal volume per joule (**a**) and removal volume per second (**b**) at various combinations of the scanning speed and repetition rate. The pulse energy is 8 μJ, and the peak laser fluence is 0.416 J/cm^2^. The number of scans is 50.

As shown in Figure 12c,d, low repetition rates resulted in a higher *V_e_*, while high repetition rates led to a higher *V_t_*. At the same repetition rate, as the scanning speed increased, the *V_e_* and *V_t_* increased simultaneously, which can be seen in Figure 13. It should be noted that a combination of a high scanning speed and a low repetition rate may result in a low overlap ratio (e.g., η≤0.592), the morphology of the scribed groove was discontinuous, and the corresponding *V_e_* and *V_t_* were treated with zero value.

At low repetition rates, the input laser energy is not overly redundant and is well coupled with the target material; as a result, a higher energy utilization efficiency can be obtained. At high repetition rates, the reasons for low energy utilization efficiency include: First, due to the high overlap ratio, the laser energy is highly redundant. Second, high repetition rates led to enhanced coupling between the subsequent pulse and the laser-induced plasma, and the plasma shielding effect and the scattering effect induced by the clusters/particles became prominent, greatly reducing the deposited laser energy. Third, at high overlap ratios, the materials underwent severe melting and re-solidification, which may cause the material to not be effectively removed, resulting in a waste of laser energy.

At high repetition rates, a higher ablation efficiency can generally be obtained. This is because a high overlap ratio can still be guaranteed in a certain speed range (e.g., 25 mm/s–500 mm/s); with the increase in the scanning speed, the heat accumulation effect is still significant, and the thermal ablation mechanism dominates. As a result, a high ablation efficiency is to be expected.

**Figure 14:**
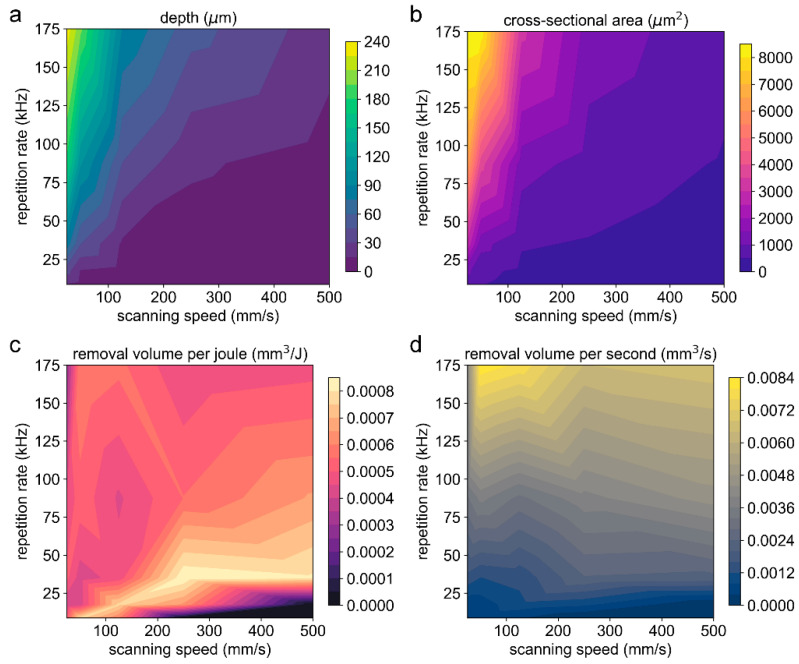
The contour map for the groove depth (**a**), cross-sectional area (**b**), removal volume per joule (**c**), and removal volume per second (**d**) at various combinations of the scanning speed and repetition rate. The pulse energy is 80 μJ, and the peak laser fluence is 4.16 J/cm^2^. The number of scans is 50.

Figure 14a–d show the contour map for the groove depth, cross-sectional area, removal volume per joule (*V_e_*), and removal volume per second (*V_t_*), respectively, at various combinations of the scanning speed and repetition rate with a pulse energy of 80 μJ. Similar to the case of 8 μJ, the maximum depth and the maximum cross-sectional area were obtained simultaneously at a combination of a high repetition rate and a low scanning speed.

**Figure 15:**
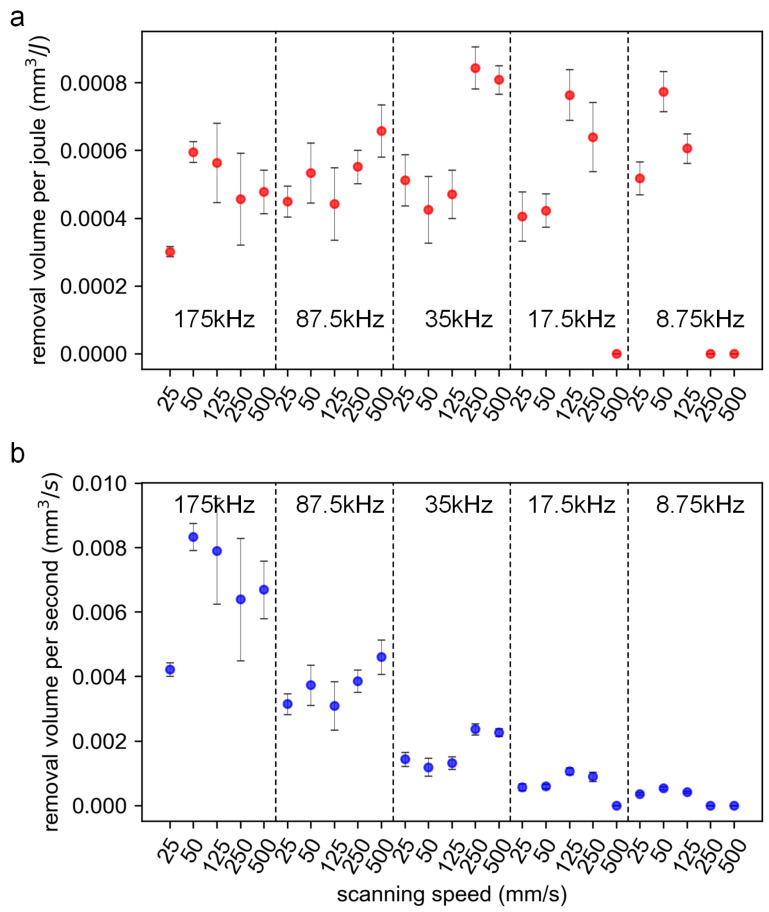
The removal volume per joule (**a**) and removal volume per second (**b**) at various combinations of the scanning speed and repetition rate. The pulse energy is 80 μJ, and the peak laser fluence is 4.16 J/cm^2^. The number of scans is 50.

However, the relationship among the *V_e_* (or *V_t_*), repetition rate, and scanning speed did not show obvious regularity and was a bit disorganized when high pulse energy was used, as shown in Figure 14c,d, which may be related to perturbations induced by the strong thermal ablation during the laser scribing. There was a trend that low repetition rates resulted in a higher *V_e_*, and high repetition rates led to a higher *V_t_*. In addition, a higher *V_e_* and *V_t_* could also be obtained at some specific combinations, i.e., 175 kHz and 50 mm/s (Figure 15). At a high repetition rate, a low scanning speed contributes to a high overlap ratio, and thermal ablation dominates. As the scanning speed increases, the overlap ratio decreases, the thermal effect is weakened, the driving pressure inside the molten layer is insufficient, and the molten layer cannot be effectively removed; as a result, the effective removal volume decreases. This further confirms the perturbation of thermal ablation at high pulse energies.

**Figure 16:**
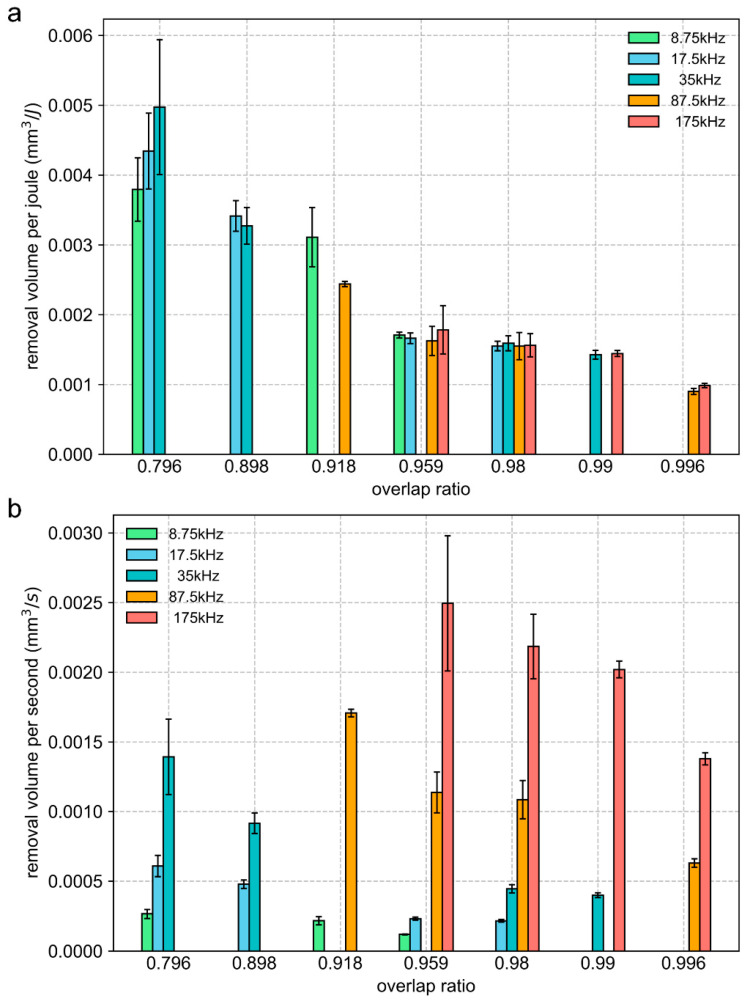
The removal volume per joule (**a**) and removal volume per second (**b**) at various overlap ratios. The pulse energy is 8 μJ and the peak laser fluence is 0.416 J/cm^2^. The number of scans is 50.

Figure 16 shows the removal volume per joule (*V_e_*) and removal volume per second (*V_t_*) at different overlap ratios with a pulse energy of 8 μJ. An interesting phenomenon is that, as the overlap ratio increases, the energy utilization efficiency decreases, which may be related to the plasma shielding effect. The laser energy is absorbed by the plasma or scattered by the nanoclusters, resulting in a decrease in energy absorption. It is worth noting that the energy utilization efficiency, despite some fluctuations, is not sensitive to changes in the repetition rate at the same overlap ratio, which is consistent with other pulse energies (refer to the Supplementary Materials, Figures S1–S4). At the same overlap ratio with the same laser fluence, the laser energy is deposited at the same space separation (i.e., the same accumulated laser fluence *H_acc_*), triggering a similar thermal process and resulting in a consistent energy utilization efficiency. From these experimental data, one can conclude that there is a strong correlation between energy utilization efficiency and the overlap ratio.

For ablation efficiency, at the same overlap ratio with the same laser fluence, the ablation efficiency increases as the repetition rate increases. There was no significant correlation between ablation efficiency and the overlap ratio. At the same overlap ratio, a high repetition rate leads to a high scanning speed, so the required time is reduced, and the resulting removal volume per second (*V_t_*) is increased.

## 4. Conclusions

In this paper, the temperature evolution at various combinations of repetition rate and scanning speed was investigated based on a two-temperature model involving a moving laser source. Experimentally, the effects of the pulse energy, repetition rate, scanning speed, and overlap ratio on ablation quality and productivity were investigated. Special attention was paid to the analysis of the groove morphology, groove depth, cross-sectional area, removal volume per joule (*V_e_*), and removal volume per second (*V_t_*). Some meaningful conclusions have been drawn as follows.

(1) Pulse energy determines the peak thermal equilibrium temperature between the electron and lattice. When the equilibrium temperature far exceeds the critical temperature, obvious thermal ablation occurs in the groove, and the processing quality deteriorates. In this experiment, a pulse energy smaller than 40 μJ is preferred to improve the surface quality.
(2) As indicated by the simulation model, the temperature evolution at the initial position influenced by subsequent pulses is strongly associated with the overlap ratio. The thermal effect of subsequent pulses is significantly weakened when the overlap ratio is 0.796 and is negligible when the overlap ratio is 0.184.
(3) At the same laser fluence, the groove morphology has a stable correspondence with the overlap ratio, which is controllable by selecting the overlap ratio.
(4) For energy utilization efficiency, due to its insensitivity to changes in the repetition rate at the same overlap ratio, the number of experimental samples can be reduced, and the cost of the test can be decreased in determining the optimal overlap ratio. For ablation efficiency, at the same overlap ratio with the same laser fluence, the ablation efficiency increases with the increase in the repetition rate.

In conclusion, the following processing strategy can be derived from the research: Firstly, pulse energy determines the peak thermal equilibrium temperature between the electron and lattice; when the equilibrium temperature exceeds the critical temperature, obvious thermal ablation occurs, so choosing the laser energy corresponding to an equilibrium temperature less than the critical temperature helps to improve the surface quality. Then, the overlap ratio can be used as a characteristic parameter of energy utilization efficiency, and the parameter optimization process can be greatly streamlined and shortened due to the reduced parameter dimensions. A lower overlap ratio in the range from 0.796 to 0.996 tends to result in a higher energy utilization efficiency, contributing to suppressing the thermal effect during the scribing process. Afterward, based on the determined overlap ratio, a high repetition rate contributes to boosting the ablation efficiency.

## Figures and Tables

**Table 1:** The chemical composition of the nickel-based alloy (in wt.%).

Elements	C	Al	Ti	Cr	Co	Ni	Mo	Ta	W
wt.%	6.58	2.92	4.75	12.09	8.82	Bal.	1.37	2.56	4.30

**Table 2:** Summary of the processing parameters.

Parameters	Symbol	Value	Units
Pulse energy	*E_p_*	8, 16, 40, 80, 160	μJ
Repetition rate	*f*	8.75, 17.5, 35, 87.5, 175	kHz
Scanning speed	*v*	25, 50, 125, 250, 500	mm/s
Focus diameter	d	70	μm
Number of scans	*N*	50	
Overlap ratio	η	1−v/(d·*f*)	

**Table 3:** Physical parameters of the target material for the TTM simulation.

Physical Parameter	Symbol	Value	Reference
The reflectance of the target material	*R*	0.65	[39]
Thermal conductivity of electron at *T* = 273 K	*k*_*e*,0_	91 J m^−1^ K^−1^ s^−1^	[37]
Electronic heat capacity parameter	*γ*	1065 J m^−3^ K^−2^	[38]
Thermal conductivity of lattice	*k_l_*	3.496 + 0.026733 *T_l_* − 1.11803 × 10^−5^ *T_l_*^2^ + 3.60684 × 10^−9^ *T_l_*^3^ + 8.23555 × 10^−14^ *T_l_* ^4^	[40]
Heat capacity of lattice	*C_l_*	4.1 × 10^6^ J m^−3^ K^−1^	[37]
Electron–phonon coupling strength	*g*	3.6 × 10^17^ W m^−3^ K^−1^	[41]
The optical absorption depth	*L_p_*	13.5 nm	[39]

## Data Availability

The data presented in this study are available upon reasonable request from the corresponding author.

## Supplementary Materials

The following supporting information can be downloaded at https://www.mdpi.com/article/10.3390/ma16216895/s1: Figure S1: The removal volume per joule (a) and removal volume per second (b) at various overlap ratios. The pulse energy is 16 μJ, and the peak laser fluence is 0.832 J/cm^2^. The number of scans is 50. Figure S2: The removal volume per joule (a) and removal volume per second (b) at various overlap ratios. The pulse energy is 40 μJ, and the peak laser fluence is 2.08 J/cm^2^. The number of scans is 50. Figure S3: The removal volume per joule (a) and removal volume per second (b) at various overlap ratios. The pulse energy is 80 μJ, and the peak laser fluence is 4.16 J/cm^2^. The number of scans is 50. Figure S4: The removal volume per joule (a) and removal volume per second (b) at various overlap ratios. The pulse energy is 160 μJ, and the peak laser fluence is 8.32 J/cm^2^. The number of scans is 50.
