# Supplementary material for: A Systematic Study on the Processing Strategy in Femtosecond Laser Scribing via a Two-Temperature Model
Source: Materials (Basel). 2023 Oct 27;16(21):6895. doi: 10.3390/ma16216895 (PMC10647803; doi:10.3390/ma16216895)
Supplement: Supplementary file 1 [file materials-16-06895-s001.zip › materials-2639931-supplementary.pdf]

Supplementary Materials for

A Systematic Study on the Processing Strategy in Femtosecond Laser Scribing via a Two-Temperature Model

Rujia Wang[1,2], Yufeng Wang[1,2,3], Yong Yang[1,2,3], Shuowen Zhang[1,2], Yunfeng Liu[4], Jianhua Yao[4], Wenwu Zhang[1,2,3,*]

[1] Ningbo Institute of Materials Technology and Engineering, Chinese Academy of Sciences, Ningbo 315201, China; rujiawang@nimte.ac.cn (R.W.); wangyufeng@nimte.ac.cn (Y.W.); yangyong1994@nimte.ac.cn (Y.Y.); zhangsw_edu@163.com (S.Z.)

[2] Zhejiang Key Laboratory of Aero Engine Extreme Manufacturing Technology, Ningbo 315201, China

[3] University of Chinese Academy of Sciences, Beijing 100049, China

[4] College of Mechanical Engineering, Zhejiang University of Technology, Hangzhou 310023, China;

liuyf76@126.com (Y.L.); laser@zjut.edu.cn (J.Y.)

* Correspondence: zhangwenwu@nimte.ac.cn

Figures

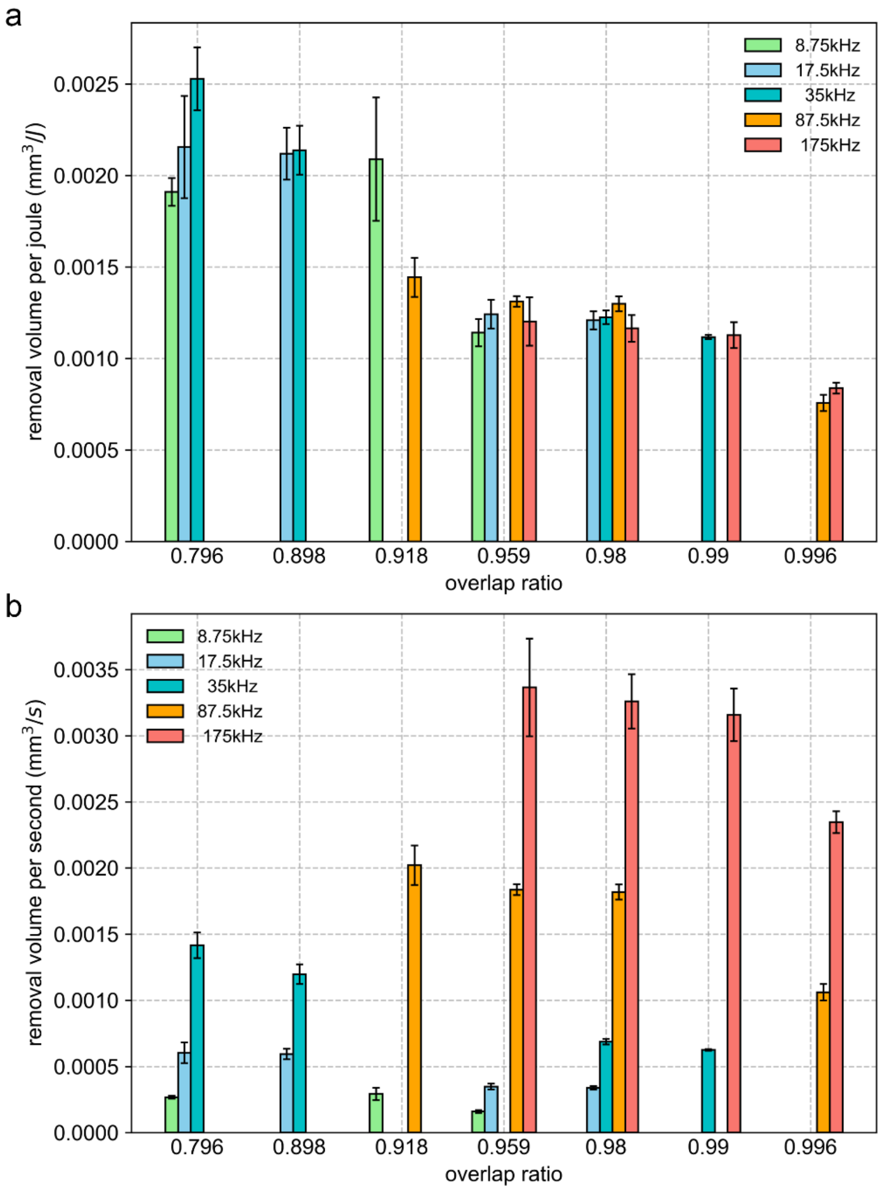

Figure S1. The removal volume per joule (a) and removal volume per second (b) at various overlap ratios. The pulse energy is 16 μJ and the peak laser fluence is 0.832 J/cm^2. The number of scans is 50.

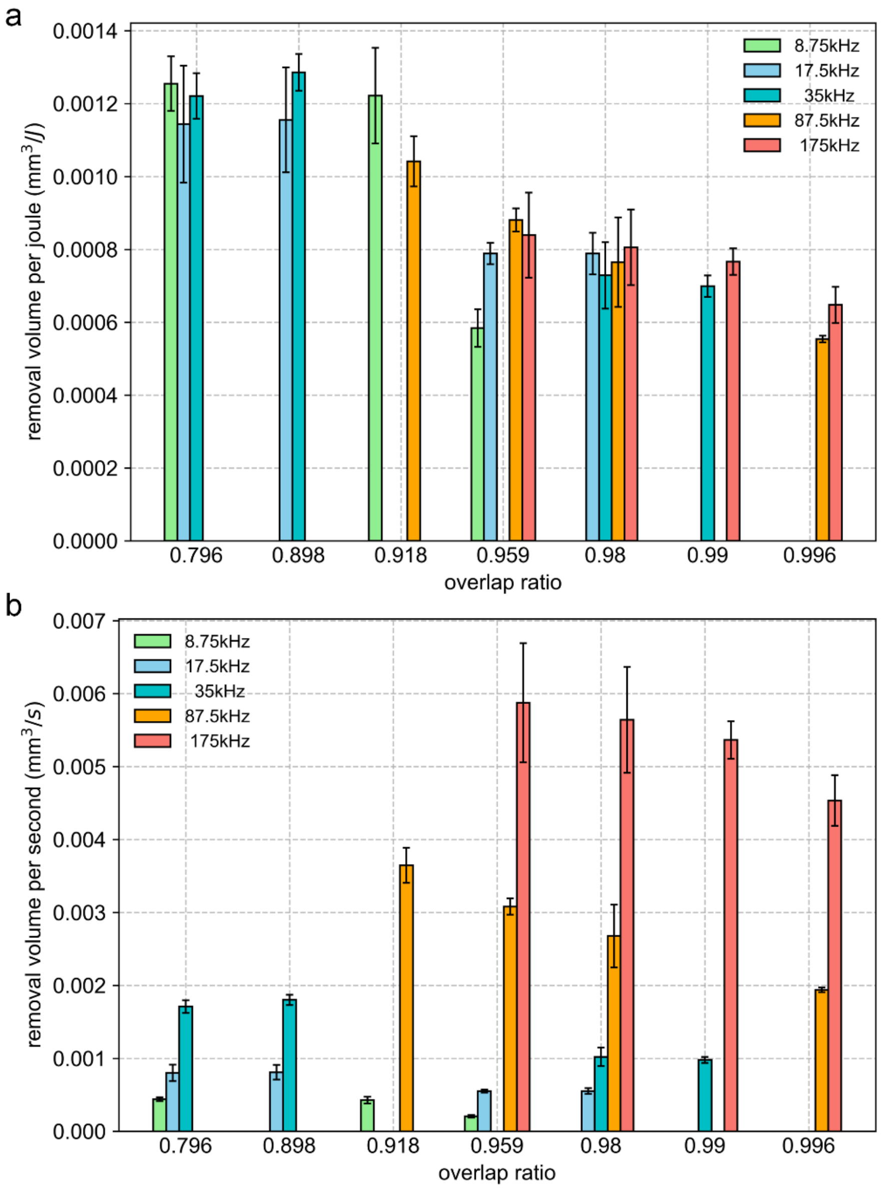

Figure S2. The removal volume per joule (a) and removal volume per second (b) at various overlap ratios. The pulse energy is 40 μJ and the peak laser fluence is 2.08 J/cm^2. The number of scans is 50.

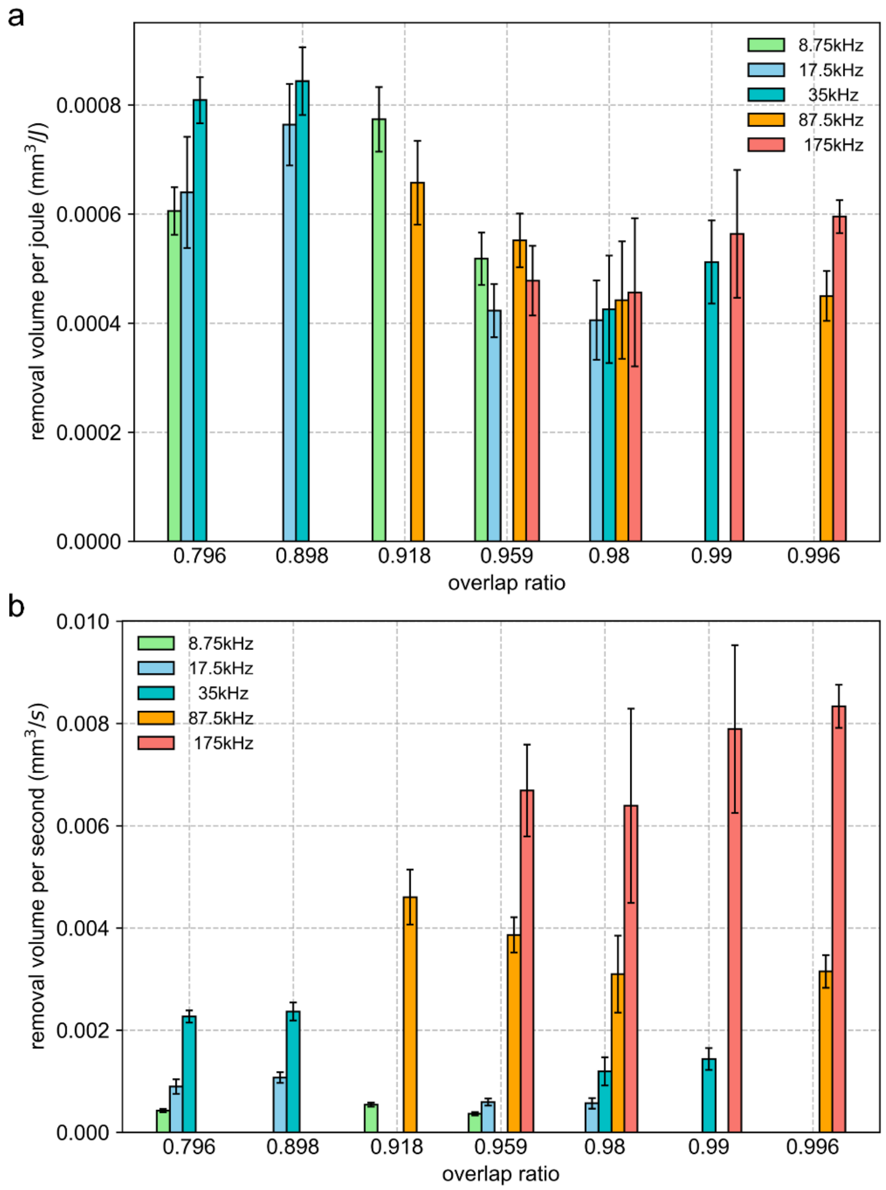

Figure S3. The removal volume per joule (a) and removal volume per second (b) at various overlap ratios. The pulse energy is 80 μJ and the peak laser fluence is 4.16 J/cm^2. The number of scans is 50.

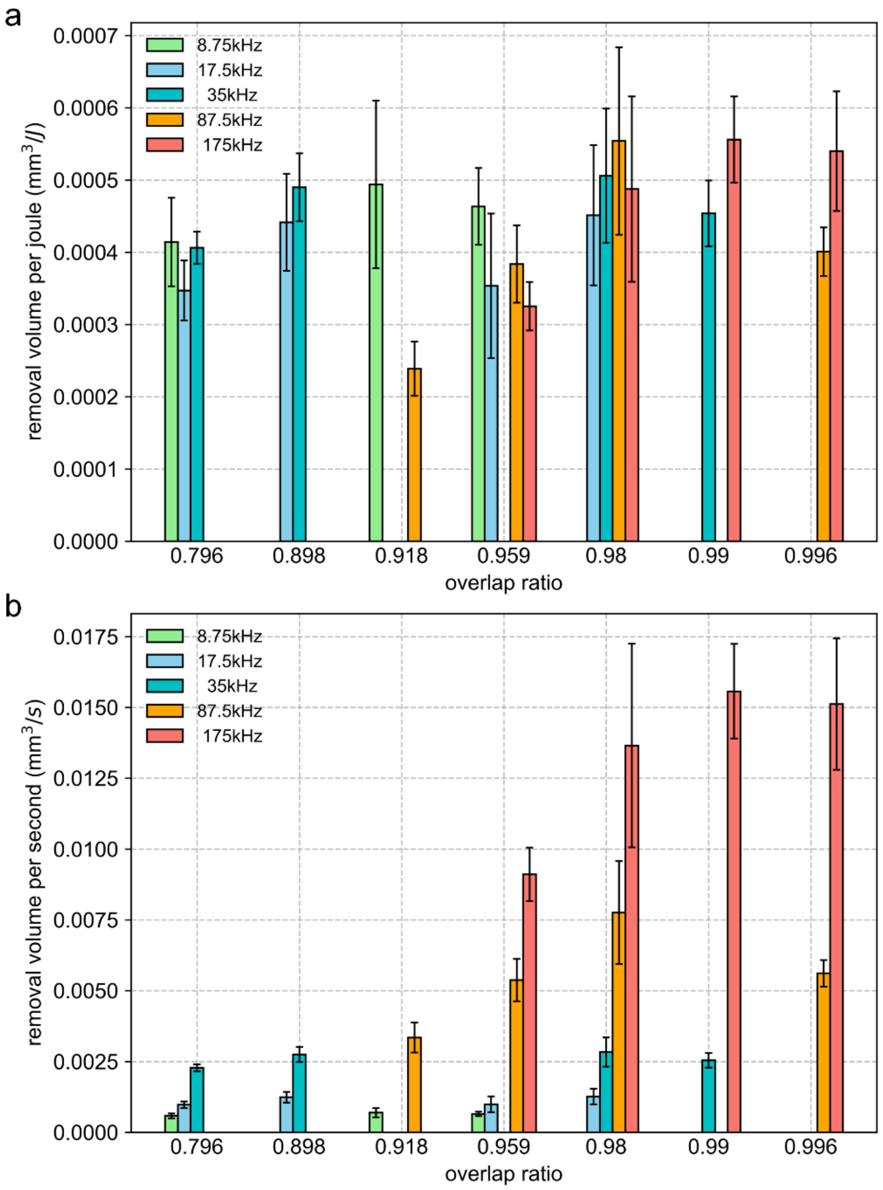

Figure S4. The removal volume per joule (a) and removal volume per second (b) at various overlap ratios. The pulse energy is 160 μJ and the peak laser fluence is 8.32 J/cm^2. The number of scans is 50.
